# Predictors and Outcomes of Right Ventricular Dysfunction in Patients Admitted to the Medical Intensive Care Unit for Sepsis—A Retrospective Cohort Study

**DOI:** 10.3390/jcm14155423

**Published:** 2025-08-01

**Authors:** Raksheeth Agarwal, Shreyas Yakkali, Priyansh Shah, Rhea Vyas, Ankit Kushwaha, Ankita Krishnan, Anika Sasidharan Nair, Balaram Krishna Jagannayakulu Hanumanthu, Robert T. Faillace, Eleonora Gashi, Perminder Gulani

**Affiliations:** 1 Department of Medicine, Jacobi Medical Center/Albert Einstein College of Medicine, 1400 Pelham Parkway South, Bronx, NY 10461, USA; 2 Department of Pulmonary and Critical Care Medicine, Jackson Madison County General Hospital, Jackson, TN 38305, USA; 3 Department of Medicine, Critical Care Division, Cleveland Clinic, Cleveland, OH 44195, USA; 4 Department of Cardiovascular Medicine, University of Pennsylvania, Philadelphia, PA 19104, USA

**Keywords:** critical care, sepsis, right ventricular dysfunction, transthoracic echocardiography

## Abstract

**Background**: Right ventricular (RV) dysfunction is associated with poor clinical outcomes in critically ill sepsis patients, but its pathophysiology and predictors are incompletely characterized. We aimed to investigate the predictors of RV dysfunction and its outcomes in sepsis patients admitted to the intensive care unit (ICU). **Methods**: This is a single-center retrospective cohort study of adult patients admitted to the ICU for sepsis who had echocardiography within 72 h of diagnosis. Patients with acute coronary syndrome, acute decompensated heart failure, or significant valvular dysfunction were excluded. RV dysfunction was defined as the presence of RV dilation, hypokinesis, or both. Demographics and clinical outcomes were obtained from electronic medical records. **Results**: A total of 361 patients were included in our study—47 with and 314 without RV dysfunction. The mean age of the population was 66.8 years and 54.6% were females. Compared to those without RV dysfunction, patients with RV dysfunction were more likely to require mechanical ventilation (63.8% vs. 43.9%, *p* = 0.01) and vasopressor support (61.7% vs. 36.6%, *p* < 0.01). On multivariate logistic regression analysis, increasing age (OR 1.03, 95% C.I. 1.00–1.06), a history of HIV infection (OR 5.88, 95% C.I. 1.57–22.11) and atrial fibrillation (OR 4.34, 95% C.I. 1.83–10.29), and presence of LV systolic dysfunction (OR 14.40, 95% C.I. 5.63–36.84) were independently associated with RV dysfunction. Patients with RV dysfunction had significantly worse 30-day survival (Log-Rank *p* = 0.023). On multivariate Cox regression analysis, older age (HR 1.02, 95% C.I. 1.00–1.04) and peak lactate (HR 1.16, 95% C.I. 1.11–1.21) were independent predictors of 30-day mortality. **Conclusions**: Among other findings, our data suggests a possible association between a history of HIV infection and RV dysfunction in critically ill sepsis patients, and this should be investigated further in future studies. Patients with evidence of RV dysfunction had poorer survival in this population; however this was not an independent predictor of mortality in the multivariate analysis. A larger cohort with a longer follow-up period may provide further insights.

## 1. Introduction

Sepsis is a complex and life-threatening condition characterized by a dysregulated host response to infection, leading to widespread inflammation, tissue damage, and organ dysfunction [1]. During the state of septic shock cytokines, complement cascade activation, molecular patterns (endogenous and pathogen driven), oxidative stress, altered nitric oxide metabolism, mitochondrial dysfunction, abnormal calcium movement within cells, myocyte apoptosis, and autonomic dysregulation lead to myocardial depression [2]. Despite an increase in the coronary perfusion, the aforementioned mechanisms lead to reversible systolic and/or diastolic dysfunction of one or both ventricles, which has been extensively studied in the context of left ventricular (LV) dysfunction [3]. However, right ventricular (RV) dysfunction in sepsis remains less well understood, despite its significant impact on patient outcomes.

Right ventricular (RV) dysfunction is an increasingly recognized clinical concern, particularly in patients with sepsis. In the short term, RV dysfunction can lead to accelerated left ventricular impairment, hemodynamic instability, progression of the underlying condition, and higher mortality risk [4]. Over the long term, it contributes to multi-organ failure and the development of chronic congestive heart failure, further elevating the risk of mortality [5]. Plasma biomarkers and imaging studies can aid in the diagnosis of RV dysfunction. Elevations in troponin and B-type natriuretic peptide levels are often observed in septic patients with RV dysfunction and can signal the need for further testing [6]. Transthoracic echocardiography remains the cornerstone of RV dysfunction diagnosis. Recent guidelines by the American Society of Echocardiography recommend comprehensive assessment of the right ventricle, including RV fractional area change, RV free wall longitudinal strain, and 3D RV ejection fraction [7].

Recent studies have highlighted the prevalence of RV dysfunction in septic patients, with estimates suggesting it affects approximately 40–50% of this population [8]. The pathophysiology of RV dysfunction in sepsis is multifactorial, involving myocardial injury, increased pulmonary vascular resistance, and altered hemodynamics. These changes can lead to decreased RV contractility and impaired cardiac output, contributing to poor clinical outcomes, including increased mortality and prolonged intensive care unit (ICU) stays [6]. Right ventricular dysfunction is associated with worse 30-day survival in severely and critically ill COVID-19 patients [9]. The prognostic significance of RV dysfunction in sepsis has been increasingly recognized. For instance, a recent study demonstrated that RV dysfunction is associated with a threefold increase in 28-day mortality in septic patients [8]. Additionally, isolated RV dysfunction was found to be an independent risk factor for short-term mortality in patients with septic shock [10]. These findings underscore the importance of standardized assessment and early detection of RV dysfunction to optimize outcomes in patients with sepsis.

Despite its clinical importance, the predictors of RV dysfunction in sepsis are not well defined, necessitating further research to elucidate these factors and improve patient management. This study aims to investigate the factors associated with RV dysfunction and its outcomes in sepsis patients admitted to the ICU, providing insights that could enhance prognostic assessments and therapeutic strategies.

## 2. Methods

### 2.1. Study Population and Design

This was a single-center retrospective cohort study conducted at a large municipal hospital in the Bronx, New York. All consecutive adult patients admitted to the medical ICU between 1 January 2016 and 31 December 2017 with a diagnosis of sepsis who had a transthoracic echocardiogram (TTE) within 72 h of admission were included in this study. Patients with acute coronary syndrome, acute decompensated heart failure, or significant valvular dysfunction were excluded. This study was approved by the IRB at Albert Einstein College of Medicine (IRB No. 2018-8773) on 12 February 2018, and requirement for informed consent was waived due to the retrospective nature of the study.

### 2.2. Data Collection

All data was collected via the hospital’s secure electronic medical record system. Demographic information, substance use history, and past medical history were collected, including conditions such as hypertension, diabetes, hyperlipidemia, cerebrovascular accident, cirrhosis, end-stage renal disease (ESRD), human immunodeficiency virus (HIV) infection, chronic obstructive pulmonary disease (COPD), clinically diagnosed congestive heart failure (CHF), coronary artery disease (CAD), peripheral artery disease (PAD), venous thromboembolism (VTE), and atrial fibrillation. Results from laboratory and microbiological investigations (including basic metabolic panel, complete blood count, peak lactate during admission, hemoglobin A1c, and culture positivity including the micro-organisms) were also noted. The collected data were used to calculate the Sequential Organ Failure Assessment (SOFA) and Acute Physiology and Chronic Health Evaluation (APACHE) II scores at the time of admission. Additional admission information, including vital signs, occurrence of pulmonary embolism or acute respiratory distress syndrome (ARDS), and the need for mechanical ventilation, vasopressor support, or renal replacement therapy, was also recorded. Outcomes of hospitalization including 30-day survival and in-hospital mortality were also extracted.

### 2.3. Transthoracic Echocardiography

In addition to the data outlined above, results of TTE assessments during admission were recorded. The TTE data extracted for this study included LV and RV function, LV ejection fraction (LVEF), and the presence of regional wall motion abnormalities (RWMAs). LVEF was either measured quantitatively using the Simpson’s biplane method or estimated visually when image quality did not allow for accurate quantification. An LVEF < 50% was used to define LV systolic dysfunction. Right ventricular dysfunction was defined as the presence of either RV systolic dysfunction (as denoted by an abnormal tricuspid annular plane systolic excursion (TAPSE) of <17 mm) or RV dilation (as defined by an RV basal diameter > 41 mm or by visual estimation).

### 2.4. Statistical Analysis

All statistical analyses were conducted using SPSS Statistics 26 (IBM corporations, Armonk, NY, USA) and RStudio Version 2025.05.1+513 (Posit PBC, Boston, MA, USA). Normality of data was determined by the Shapiro–Wilk test. Continuous data were compared using the Student’s *t* test when normally distributed or by the Mann–Whitney’s U test when non-normal. Categorical data were compared using the Chi-Square test or Fisher’s Exact test where appropriate. Multivariate logistic regression analysis was conducted to find the factors independently associated with RV dysfunction. This model incorporated demographic factors (age, sex, body mass index), cardiovascular risk factors (hypertension, diabetes, hyperlipidemia), and other comorbidities and admission variables that were different between the two groups. A Kaplan–Meier curve was created, and univariate and multivariate Cox regression analyses were conducted for survival analysis. We performed the univariate Cox regression analysis with all demographic factors, comorbidities, admission data, and admission outcome variables. Subsequently, we incorporated the variables that were significant on univariate analysis into a multivariate Cox regression model to identify independent predictors of mortality. To assess the proportional hazards assumption of the Cox regression model, we computed the Schoenfeld test on RStudio. Both individual covariates and the global test were examined. A *p*-value of <0.05 was considered significant.

## 3. Results

A total of 361 patients were included in this study, 47 with and 314 without evidence of RV dysfunction. The mean age of the population was 66.8, and 54.6% were females. Population characteristics including demographics, social history, past medical history, and admission information and outcomes can be seen in Table 1.

A multivariate logistic regression analysis was performed to ascertain the effects of covariates on the likelihood that subjects have RV dysfunction. This multivariate logistic regression model was statistically significant (χ^2^ = 67.49, *p* < 0.001) and correctly classified 86.9% of cases. The Hosmer–Lemeshow test showed a non-significant result (χ^2^ = 6.702, *p* = 0.569), indicating that the model fits the data well. Increasing age, a history of HIV infection and atrial fibrillation, and presence of LV systolic dysfunction were independently associated with RV dysfunction in this model (Table 2).

**Figure 1:**
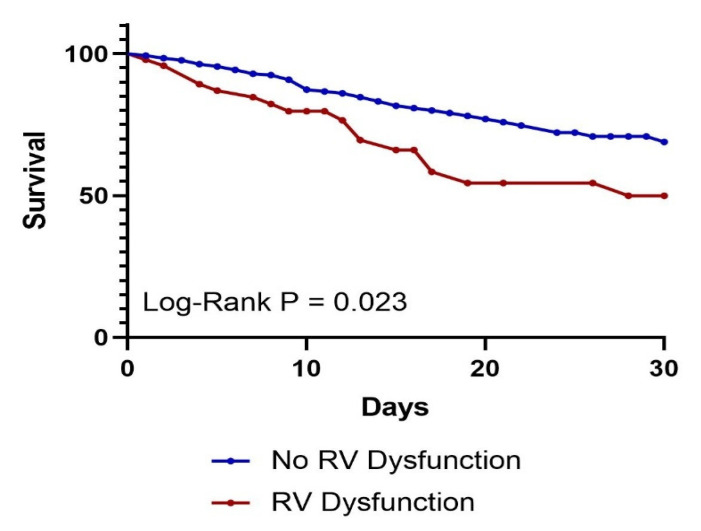
Kaplan–Meier 30-day survival curve by presence of RV dysfunction. *p* = 0.023.

Next, we performed survival analysis for 30-day survival. The Kaplan–Meier curve (Figure 1) showed that individuals with RV dysfunction had significantly poorer survival compared to those without RV dysfunction (Log-Rank *p* = 0.023).

This was corroborated by univariate Cox regression analysis, which identified increasing age, lower mean arterial pressure, acute kidney injury (AKI), higher APACHE and SOFA scores, vasopressor or ventilator requirement, and presence of RV dysfunction and shock as predictors of 30-day mortality. When these variables were analyzed in a multivariate Cox regression model, only increasing age and peak lactate emerged as independent predictors of 30-day mortality (Table 3). The proportional hazards assumption was evaluated using Schoenfeld residuals. None of the covariates showed significant deviations from proportionality (all *p* > 0.05). The global test for the model also confirmed that the assumption held (χ^2^ = 7.81, *p* = 0.65).

## 4. Discussion

Our study found that increasing age, LV systolic dysfunction, and a history of HIV infection or atrial fibrillation were significantly associated with RV dysfunction in critically ill sepsis patients admitted to the ICU (Table 2). While Kaplan–Meier analysis (Figure 1) demonstrated worse short-term survival among patients with RV dysfunction, this did not remain an independent predictor of 30-day mortality in multivariate Cox regression analysis (Table 3). Nonetheless, patients with RV dysfunction more frequently required vasopressor support and mechanical ventilation compared to those without RV dysfunction (Table 1).

These findings are consistent with existing literature on RV dysfunction in critical illness. Right ventricular (RV) dysfunction is a significant issue in the management of critically ill patients. Acute increases in RV afterload—such as those caused by pulmonary embolism, acute respiratory distress syndrome (ARDS), or mechanical ventilation with high airway pressures—are key risk factors. Chronic conditions like CODP may also contribute by chronically elevating pulmonary vascular resistance [11]. In addition to these mechanical stressors, inflammatory biomarkers and concurrent left ventricular (LV) dysfunction have emerged as predictors of RV dysfunction in acute illnesses, including COVID-19 [9]. Several prognostic markers have been associated with worse outcomes in patients with RV dysfunction. These include advanced age, elevated N-terminal pro–B-type natriuretic peptide (NT-proBNP), increased creatinine levels, and systolic blood pressure below 100 mmHg. Outcomes are particularly poor in those with severe RV dysfunction, with reported one- and five-year survival rates of 61% and 35%, respectively [12]. Among patients with sepsis, RV dysfunction has been consistently associated with increased short-term and long-term mortality, highlighting its importance as both a diagnostic and prognostic marker in this population [13]. Due to the complex pathophysiology of sepsis-related RV dysfunction, evidence on disease-modifying therapies in the acute setting remains limited. Current management focuses on controlling the underlying infection, optimizing fluid resuscitation, and providing vasopressor support. Select agents such as milrinone and low-dose vasopressin—which may reduce pulmonary vascular resistance—can offer additional hemodynamic benefits in patients with RV dysfunction. However, their use should be individualized and guided by comprehensive clinical and hemodynamic assessment [6].

Our data indicate that patients with HIV infection had six times greater odds of developing RV dysfunction (OR 5.88), independent of other covariates. While the association between HIV and pulmonary arterial hypertension is well established [14], literature on its relationship with RV dysfunction remains limited. In the Multicenter AIDS Cohort Study, HIV seropositivity was associated with RV dilation and systolic dysfunction, likely due to adverse cardiac remodeling from elevated pulmonary artery pressures and increased afterload [15]. Another study observed RV dysfunction in HIV-positive patients without evidence of pulmonary hypertension, suggesting additional mechanisms such as direct myocardial inflammation or HIV-related infiltration may also contribute [16]. The HIV prevalence in our cohort was 6.2%, substantially exceeding the estimated U.S. prevalence of under 1% (approximately 1.2 million in 2022) [17], which increased the statistical power to detect this association. To our knowledge, this is one of the few studies that highlights a significant association between HIV infection and RV dysfunction in the context of sepsis, warranting further mechanistic exploration.

Right ventricular (RV) dysfunction was associated with worse clinical outcomes in our study. Kaplan–Meier analysis (Figure 1; Log-Rank *p* = 0.023) demonstrated a significantly increased risk of 30-day mortality in patients with RV dysfunction. This finding is consistent with a meta-analysis of 1373 patients, which reported that RV dysfunction was independently associated with both short- and long-term mortality [13]. However, our study did not identify RV dysfunction as an independent predictor of 30-day mortality in multivariate Cox regression (Table 3). A potential explanation is the lower incidence of RV dysfunction in our cohort (13% vs. 35%), which may have limited our statistical power to detect a significant association.

In their cohort of critically ill sepsis patients admitted to the ICU, Hiraiwa et al. demonstrated that RV dysfunction correlated with elevated lactate levels—indicative of circulatory insufficiency—and an increased incidence of fatal arrhythmias, offering potential mechanistic insights [18]. In sepsis, RV dysfunction may result from a combination of systemic inflammation, increased preload due to fluid resuscitation, and increased afterload from ARDS and positive-pressure ventilation strategies [9,18]. The failing right ventricle may then be unable to sustain adequate cardiac output, contributing to circulatory insufficiency and shock state. In our cohort, this was reflected by a higher proportion of patients with RV dysfunction requiring vasopressor support (Table 1). Cardiogenic shock may further predispose patients to complications such as malignant ventricular arrhythmias [18].

Our study identified increasing age as an independent predictor of 30-day mortality, consistent with findings from recent studies on critically ill sepsis patients in the ICU [19,20,21,22]. Michels et al. demonstrated that increasing age was a predictor of mortality independent of age-related comorbidities and disease severity. Their analysis of blood biomarkers revealed that older patients exhibited reduced endothelial activation and decreased expression of genes involved in innate and adaptive immunity and cytokine signaling [22]. These findings suggest that immunosenescence and other age-related physiological changes may contribute to poorer outcomes in older sepsis patients. We demonstrated an independent association between increasing age and RV dysfunction in our cohort (Table 2). This raises the hypothesis that aging may increase the vulnerability of the RV in sepsis, thereby contributing to worse outcomes including mortality. In addition to increasing age, peak lactate was independently associated with 30-day mortality in our cohort, a finding well supported by prior literature [23]. This association underpins the emphasis on lactate measurement in the Surviving Sepsis Campaign guidelines [24].

## 5. Limitations and Future Perspectives

This study has several limitations that should be considered when interpreting the findings. First, its retrospective design limited data availability and introduced potential bias. Critical variables—such as the presence of pulmonary hypertension (assessed via transthoracic echocardiography or right-heart catheterization), cause of death, and incidence of malignant arrhythmias—were not available, limiting our ability to determine whether pulmonary hypertension mediated the association between HIV and RV dysfunction, or whether RV dysfunction contributed to malignant arrhythmias. Furthermore, key HIV-related variables such as antiretroviral therapy status, CD4 counts, and immune function were unavailable, limiting our ability to explore associations between HIV disease control, sepsis etiology and source, and RV dysfunction. Second, the single-center nature of the study may limit the generalizability of our findings and reduce statistical power, increasing the risk of type 2 error and external validity. Third, only a single transthoracic echocardiogram was available for each patient, preventing us from determining whether RV dysfunction was pre-existing or developed during the ICU stay. As a result, we cannot establish whether patients in this study had sepsis-associated RV dysfunction, RV involvement due to septic cardiomyopathy, or pre-existing RV dysfunction.

Furthermore, our study used TAPSE and RV dilation as the sole echocardiographic markers to define RV dysfunction. While TAPSE is a widely accepted and easily obtainable metric, it reflects only the longitudinal motion of the RV free wall and may not adequately capture global or radial dysfunction. As highlighted in a recent state of the art review, TAPSE can remain within the normal range even in the presence of significant right ventricular impairment, particularly in pressure overloaded states such as pulmonary hypertension [25]. RV dilation, similarly, may not necessarily imply systolic dysfunction and can be influenced by chronic remodeling. A more comprehensive assessment, incorporating parameters like RV fractional area change, RV free wall longitudinal strain, and 3D RV ejection fraction, is recommended by recent guidelines from the American Society of Echocardiography [7] but was not available in our dataset. In addition, information on tricuspid regurgitant velocity, right atrial pressure, and left ventricular diastolic function was not reliably obtainable, limiting our ability to estimate pulmonary vascular resistance and characterize RV afterload. Future prospective studies should incorporate comprehensive RV assessment tools and perform serial evaluations to better characterize the pathophysiology and predictors of RV dysfunction in critically ill septic patients, including those with HIV.

Despite this limitation, our findings highlight that RV dysfunction is frequently observed in septic ICU patients and is associated with several clinical and hemodynamic factors. Consistent with prior studies, we also demonstrate that the presence of RV dysfunction may negatively impact prognosis, underscoring its potential clinical relevance regardless of etiology.

## 6. Conclusions

We identified several factors potentially associated with RV dysfunction in critically ill sepsis patients admitted to the ICU, which have been corroborated by other studies. Notably, a history of HIV infection was associated with RV dysfunction in our cohort—a novel finding that warrants further investigation. Given the retrospective nature of this study, these findings should be interpreted cautiously, and prospective studies are needed to validate them. We also found that increasing age was independently associated with both RV dysfunction and 30-day mortality, raising the hypothesis that aging may increase the vulnerability of the right ventricle to dysfunction in the setting of sepsis. Our findings reinforce that RV dysfunction is associated with worse clinical outcomes, possibly by contributing to circulatory insufficiency in the context of sepsis—a condition already marked by significant hemodynamic and cardiovascular compromise. Identifying high-risk subgroups, such as older adults and those with HIV, may help guide more intensive hemodynamic monitoring and individualized management. This study provides a foundation for future research into the pathophysiology, prognostic implications, and therapeutic targeting of RV dysfunction in sepsis.

## Figures and Tables

**Table 1:** Population characteristics.

	Entire Cohort N = 361		No RV Dysfunction N = 314		RV Dysfunction N = 47		*p*-Value
Demographics, Social, and Past Medical History							
Age, mean (SD)	66.79	16.51	66.25	16.51	70.38	16.20	0.083 ^†^
Females, N (%)	197	54.6%	175	55.7%	22	46.8%	0.252
BMI, mean (SD)	29.11	15.85	29.23	16.57	28.28	9.81	0.919 ^†^
Hypertension, N (%)	236	65.6%	201	64.2%	35	74.5%	0.168
Diabetes, N (%)	148	41.2%	125	40.1%	23	48.9%	0.249
Hyperlipidemia, N (%)	125	34.8%	108	34.6%	17	36.2%	0.835
Current smoking, N (%)	77	22.1%	68	22.4%	9	19.6%	0.661
Use of cocaine, N (%)	21	6.1%	17	5.7%	4	8.7%	0.502 ^‡^
Use of alcohol, N (%)	58	16.6%	53	17.5%	5	10.9%	0.261
History of CVA, N (%)	81	22.5%	74	23.6%	7	14.9%	0.180
Cirrhosis, N (%)	33	9.2%	29	9.3%	4	8.5%	1.000 ^‡^
ESRD, N (%)	29	8.1%	22	7.1%	7	14.9%	0.082 ^‡^
HIV infection, N (%)	22	6.2%	16	5.1%	6	13.0%	0.049 ^‡^
COPD, N (%)	78	21.7%	66	21.1%	12	25.5%	0.490
CHF, N (%)	66	18.4%	52	16.7%	14	29.8%	0.030
Coronary artery disease, N (%)	62	17.2%	53	16.9%	9	19.1%	0.708
Peripheral artery disease, N (%)	26	7.2%	24	7.7%	2	4.3%	0.553 ^‡^
VTE, N (%)	72	19.9%	62	19.7%	10	21.3%	0.806
Atrial fibrillation, N (%)	65	18.1%	47	15.1%	18	38.3%	<0.001
Data on Admission							
Mean arterial pressure, mean (SD)	84.82	23.19	86.11	23.26	76.24	20.92	0.008 ^†^
Shock index at admission, mean (SD)	0.97	0.41	0.95	0.39	1.10	0.52	0.099 ^†^
AKI at admission, N (%)	254	70.4%	216	68.8%	38	80.9%	0.091
White blood cell count, mean (SD)	14.26	16.39	14.38	17.41	13.49	6.44	0.612 ^†^
HbA1c (%), mean (SD)	6.57	2.44	6.59	2.58	6.50	1.10	0.467 ^†^
APACHE-2, mean (SD)	18.99	8.25	18.61	8.25	21.49	7.87	0.037 ^†^
SOFA, mean (SD)	6.14	3.70	5.93	3.68	7.55	3.58	0.002 ^†^
LV systolic dysfunction, N (%)	43	11.9%	23	7.3%	20	42.6%	<0.01
LV ejection fraction, mean (SD)	59.88	14.24	61.77	11.84	47.32	21.08	<0.001 ^†^
Source of Sepsis by Culture Positivity							
Positive blood culture	89	24.7%	73	23.2%	16	34.0%	0.109
Positive urine culture	57	15.8%	50	15.9%	7	14.9%	0.857
Positive sputum culture	61	16.9%	51	16.2%	10	21.3%	0.390
Admission Outcomes							
Peak lactate (mmol/L), mean (SD)	4.99	4.61	4.83	4.53	5.96	5.03	0.074 ^†^
Hemodialysis required, N (%)	47	13.2%	42	13.5%	5	10.6%	0.582
Mechanical ventilation required, N (%)	168	46.5%	138	43.9%	30	63.8%	0.011
Vasopressors required, N (%)	144	39.9%	115	36.6%	29	61.7%	0.001
Developed ARDS, N (%)	20	5.6%	15	4.8%	5	10.6%	0.160 ^‡^
Developed PE, N (%)	21	5.9%	15	4.8%	6	13.0%	0.040
30-day mortality, N (%)	68	18.8%	51	16.2%	17	36.2%	0.001

^‡^ Fisher’s Exact Test. ^†^ Mann-Whitney U Test. BMI = Body Mass Index. CVA = cerebrovascular accident. ESRD = End-Stage Renal Disease. HIV = Human Immunodeficiency Virus. COPD = Chronic Obstructive Pulmonary Disease. CHF = Congestive Heart Failure. VTE = Venous Thromboembolism. AKI = Acute Kidney Injury. HbA1c = Hemoglobin A1c. APACHE = Acute Physiology and Chronic Health Evaluation. SOFA = Sequential Organ Failure Assessment. ARDS = Acute Respiratory Distress Syndrome. PE = Pulmonary Embolism.

**Table 2:** Logistic regression for right ventricular dysfunction.

Variables	Odds Ratios	*p* Value
Age	1.032 (1.001–1.064)	0.041
Female Sex	0.893 (0.427–1.869)	0.764
Body Mass Index	1.009 (0.990–1.029)	0.356
Hypertension	1.842 (0.710–4.779)	0.209
Diabetes	1.077 (0.490–2.369)	0.853
Hyperlipidemia	0.895 (0.394–2.035)	0.792
HIV Infection	5.883 (1.565–22.114)	0.009
CHF	0.590 (0.225–1.545)	0.283
Atrial Fibrillation	4.335 (1.827–10.286)	0.001
MAP at Admission	0.990 (0.971–1.008)	0.271
APACHE-2 score at Admission	0.998 (0.934–1.065)	0.942
SOFA Score at Admission	1.125 (0.975–1.299)	0.106
LV Systolic Dysfunction	14.395 (5.625–36.841)	<0.001

HIV = Human Immunodeficiency Virus. COPD = Chronic Obstructive Pulmonary Disease. CHF = Congestive Heart Failure. MAP = Mean Arterial Pressure. APACHE = Acute Physiology and Chronic Health Evaluation. SOFA = Sequential Organ Failure Assessment. LV = Left Ventricle.

**Table 3:** Univariate Cox regression analysis for 30-day mortality.

	Univariate Analysis		Multivariate Analysis	
Variables	Hazard Ratios	*p* Value	Hazard Ratios	*p* Value
Age	1.016 (1.000–1.032)	0.045	1.019 (1.002–1.036)	0.032
Female sex	1.432 (0.880–2.332)	0.148	-	-
BMI	0.993 (0.971–1.016)	0.559	-	-
Hypertension	1.021 (0.619–1.683)	0.936	-	-
Diabetes	1.066 (0.656–1.733)	0.796	-	-
Hyperlipidemia	0.631 (0.364–1.094)	0.101	-	-
Smokers	1.131 (0.634–2.017)	0.677	-	-
Cocaine users	0.171 (0.024–1.236)	0.080	-	-
Alcohol users	0.931 (0.473–1.833)	0.837	-	-
Cerebrovascular accident	1.156 (0.674–1.982)	0.598	-	-
Cirrhosis	1.443 (0.690–3.020)	0.330	-	-
ESRD	1.270 (0.581–2.779)	0.549	-	-
HIV	0.533 (0.167–1.699)	0.288	-	-
COPD	1.621 (0.951–2.761)	0.076	-	-
CHF	1.251 (0.704–2.222)	0.445	-	-
Coronary artery disease	0.908 (0.475–1.734)	0.770	-	-
Peripheral artery disease	0.997 (0.362–2.741)	0.995	-	-
Atrial fibrillation	1.549 (0.882–2.720)	0.128	-	-
MAP at admission	0.985 (0.974–0.997)	0.011	1.004 (0.992–1.017)	0.504
Shock index at admission	1.276 (0.749–2.173)	0.370	-	-
AKI at admission	1.883 (1.046–3.392)	0.035	0.860 (0.457–1.619)	0.641
Peak lactate	1.150 (1.114–1.188)	<0.001	1.161 (1.114–1.210)	<0.001
APACHE-2 at admission	1.074 (1.044–1.104)	<0.001	1.022 (0.981–1.064)	0.299
SOFA at admission	1.197 (1.125–1.274)	<0.001	1.069 (0.957–1.194)	0.235
Pressor requirement	4.243 (2.384–7.550)	<0.001	0.688 (0.144–3.285)	0.639
Ventilator requirement	4.490 (2.283–8.832)	<0.001	1.778 (0.792–3.988)	0.163
RV dysfunction	1.866 (1.076–3.233)	0.026	1.576 (0.876–2.836)	0.129
LV systolic dysfunction	0.936 (0.499–1.756)	0.837	-	-
Takotsubo cardiomyopathy	1.397 (0.666–2.931)	0.377	-	-
Developed PE	0.843 (0.306–2.317)	0.740	-	-
Developed ARDS	1.684 (0.830–3.418)	0.149	-	-
Developed shock	4.034 (2.200–7.399)	<0.001	1.943 (0.397–9.506)	0.412

BMI = Body Mass Index. ESRD = End-Stage Renal Disease. HIV = Human Immunodeficiency Virus. COPD = Chronic Obstructive Pulmonary Disease. CHF = Congestive Heart Failure. MAP = Mean Arterial Pressure. AKI = Acute Kidney Injury. APACHE = Acute Physiology and Chronic Health Evaluation. SOFA = Sequential Organ Failure Assessment. RV = Right Ventricle. LV = Left Ventricle. PE = Pulmonary Embolism. ARDS = Acute Respiratory Distress Syndrome.

## Data Availability

The data that support the findings of this study are available from the corresponding author upon reasonable request.

## References

[1] Singer M., Deutschman C.S., Seymour C.W., Shankar-Hari M., Annane D., Bauer M., Bellomo R., Bernard G.R., Chiche J.-D., Coopersmith C.M. (2016). The Third International Consensus Definitions for Sepsis and Septic Shock (Sepsis-3). JAMA.

[2] Ehrman R.R., Sullivan A.N., Favot M.J., Sherwin R.L., Reynolds C.A., Abidov A., Levy P.D. (2018). Pathophysiology, Echocardiographic Evaluation, Biomarker Findings, and Prognostic Implications of Septic Cardiomyopathy: A Review of the Literature. Crit. Care.

[3] L’Heureux M., Sternberg M., Brath L., Turlington J., Kashiouris M.G. (2020). Sepsis-Induced Cardiomyopathy: A Comprehensive Review. Curr. Cardiol. Rep..

[4] Naksuk N., Tan N., Padmanabhan D., Kancharla K., Makkar N., Yogeswaran V., Gaba P., Kaginele P., Riley D.C., Sugrue A.M. (2018). Right Ventricular Dysfunction and Long-Term Risk of Sudden Cardiac Death in Patients With and Without Severe Left Ventricular Dysfunction. Circ. Arrhythm. Electrophysiol..

[5] Sanders J.L., Koestenberger M., Rosenkranz S., Maron B.A. (2020). Right Ventricular Dysfunction and Long-Term Risk of Death. Cardiovasc. Diagn. Ther..

[6] Bansal M., Mehta A., Machanahalli Balakrishna A., Kalyan Sundaram A., Kanwar A., Singh M., Vallabhajosyula S. (2023). Right Ventricular Dysfunction in Sepsis: An Updated Narrative Review. Shock.

[7] Mukherjee M., Rudski L.G., Addetia K., Afilalo J., D’Alto M., Freed B.H., Friend L.B., Gargani L., Grapsa J., Hassoun P.M. (2025). Guidelines for the Echocardiographic Assessment of the Right Heart in Adults and Special Considerations in Pulmonary Hypertension: Recommendations from the American Society of Echocardiography. J. Am. Soc. Echocardiogr..

[8] Lanspa M.J., Cirulis M.M., Wiley B.M., Olsen T.D., Wilson E.L., Beesley S.J., Brown S.M., Hirshberg E.L., Grissom C.K. (2021). Right Ventricular Dysfunction in Early Sepsis and Septic Shock. Chest.

[9] Agarwal R., Krishnanda S.I., Yausep O.E., Nugraha R.A., Priyonugroho G., Hertine S., Wicaksono S.H., Almazini P., Zamroni D., Muliawan H.S. (2025). The Role of Neutrophil-to-Lymphocyte Ratio and Right Ventricular Dysfunction in Indonesian Patients with COVID-19: A Retrospective Cohort Study. J. Clin. Med..

[10] Kim J.-S., Kim Y.-J., Kim M., Ryoo S.M., Kim W.Y. (2020). Association between Right Ventricle Dysfunction and Poor Outcome in Patients with Septic Shock. Heart.

[11] Konstam M.A., Kiernan M.S., Bernstein D., Bozkurt B., Jacob M., Kapur N.K., Kociol R.D., Lewis E.F., Mehra M.R., Pagani F.D. (2018). Evaluation and Management of Right-Sided Heart Failure: A Scientific Statement From the American Heart Association. Circulation.

[12] Padang R., Chandrashekar N., Indrabhinduwat M., Scott C.G., Luis S.A., Chandrasekaran K., Michelena H.I., Nkomo V.T., Pislaru S.V., Pellikka P.A. (2020). Aetiology and Outcomes of Severe Right Ventricular Dysfunction. Eur. Heart J..

[13] Vallabhajosyula S., Shankar A., Vojjini R., Cheungpasitporn W., Sundaragiri P.R., DuBrock H.M., Sekiguchi H., Frantz R.P., Cajigas H.R., Kane G.C. (2021). Impact of Right Ventricular Dysfunction on Short-Term and Long-Term Mortality in Sepsis: A Meta-Analysis of 1,373 Patients. Chest.

[14] Butrous G. (2015). Human Immunodeficiency Virus-Associated Pulmonary Arterial Hypertension: Considerations for Pulmonary Vascular Diseases in the Developing World. Circulation.

[15] Doria de Vasconcellos H., Post W.S., Ervin A.-M., Haberlen S.A., Budoff M., Malvestutto C., Magnani J.W., Feinstein M.J., Brown T.T., Lima J.A.C. (2021). Associations Between HIV Serostatus and Cardiac Structure and Function Evaluated by 2-Dimensional Echocardiography in the Multicenter AIDS Cohort Study. J. Am. Heart Assoc..

[16] Çetin Güvenç R., Ceran N., Güvenç T.S., Tokgöz H.C., Velibey Y. (2018). Right Ventricular Hypertrophy and Dilation in Patients With Human Immunodeficiency Virus in the Absence of Clinical or Echocardiographic Pulmonary Hypertension. J. Card. Fail..

[17] Barry M.J., Nicholson W.K., Silverstein M., Chelmow D., Coker T.R., Davis E.M., Donahue K.E., Jaén C.R., Kubik M., US Preventive Services Task Force (2023). Preexposure Prophylaxis to Prevent Acquisition of HIV: US Preventive Services Task Force Recommendation Statement. JAMA.

[18] Hiraiwa H., Kasugai D., Ozaki M., Goto Y., Jingushi N., Higashi M., Nishida K., Kondo T., Furusawa K., Morimoto R. (2021). Clinical Impact of Visually Assessed Right Ventricular Dysfunction in Patients with Septic Shock. Sci. Rep..

[19] Bruno R.R., Wernly B., Mamandipoor B., Rezar R., Binnebössel S., Baldia P.H., Wolff G., Kelm M., Guidet B., De Lange D.W. (2021). ICU-Mortality in Old and Very Old Patients Suffering From Sepsis and Septic Shock. Front. Med..

[20] Xu C., Lv L., Hu W., Yu Z., Qian H., Chen F. (2024). Long-Term Outcomes in Older Patients with Sepsis in the ICU: A Retrospective Study. Altern. Ther. Health Med..

[21] Kotfis K., Wittebole X., Jaschinski U., Solé-Violán J., Kashyap R., Leone M., Nanchal R., Fontes L.E., Sakr Y., Vincent J.-L. (2019). A Worldwide Perspective of Sepsis Epidemiology and Survival According to Age: Observational Data from the ICON Audit. J. Crit. Care.

[22] Michels E.H.A., Butler J.M., Reijnders T.D.Y., Cremer O.L., Scicluna B.P., Uhel F., Peters-Sengers H., Schultz M.J., Knight J.C., van Vught L.A. (2022). Association between Age and the Host Response in Critically Ill Patients with Sepsis. Crit. Care.

[23] Casserly B., Phillips G.S., Schorr C., Dellinger R.P., Townsend S.R., Osborn T.M., Reinhart K., Selvakumar N., Levy M.M. (2015). Lactate Measurements in Sepsis-Induced Tissue Hypoperfusion: Results from the Surviving Sepsis Campaign Database. Crit. Care Med..

[24] Evans L., Rhodes A., Alhazzani W., Antonelli M., Coopersmith C.M., French C., Machado F.R., Mcintyre L., Ostermann M., Prescott H.C. (2021). Surviving Sepsis Campaign: International Guidelines for Management of Sepsis and Septic Shock 2021. Crit. Care Med..

[25] Hameed A., Condliffe R., Swift A.J., Alabed S., Kiely D.G., Charalampopoulos A. (2023). Assessment of Right Ventricular Function-a State of the Art. Curr. Heart Fail. Rep..

